# Knowledge and Attitudes Regarding the Vaccination of Brazilian Immigrants in Portugal: Risks When Returning to Their Country of Origin?

**DOI:** 10.3390/tropicalmed9040067

**Published:** 2024-03-22

**Authors:** Itamar P. Freitas, Ricardo P. Igreja, Maria Raquel Pacheco, Rosa Teodósio

**Affiliations:** 1 Instituto de Higiene e Medicina Tropical (IHMT), Universidade NOVA de Lisboa, Rua da Junqueira 100, 1349-008 Lisboa, Portugal; a21001047@ihmt.unl.pt (I.P.F.); rosateo@ihmt.unl.pt (R.T.); 2 Faculdade de Medicina, Universidade Federal do Rio de Janeiro, Cidade Universitária, Rio de Janeiro 21941-617, RJ, Brazil; 3 Associação para o Desenvolvimento da Medicina Tropical, Instituto de Higiene e Medicina Tropical (IHMT), Rua da Junqueira 100, 1349-008 Lisboa, Portugal; 4 Global Health and Tropical Medicine (GHTM), Instituto de Higiene e Medicina Tropical, Universidade NOVA de Lisboa, 1349-008 Lisboa, Portugal

**Keywords:** Brazilian immigrants, Portugal, vaccination, knowledge, attitudes and practices, yellow fever

## Abstract

Vaccination is one of the main advancements in public health in the prophylaxis of infectious diseases. We intend to describe the general knowledge about vaccines/vaccination among Brazilian immigrants in Portugal, characterize their attitudes toward vaccination, and describe their knowledge of the yellow fever (YF) vaccine. A cross-sectional study was conducted using a self-completion questionnaire (face-to-face or remote). A total of 542 people participated in the study; the mean age was 36.81 years; 40.1% were male; 44.8% had their 12th year of schooling; and 27.0% had resided for ≥10 years in Portugal. Regarding general knowledge about vaccination, 53.8% answered at least 6/8 questions correctly. A total of 37.1% tended to have a favorable attitude toward vaccination. Concerning traveling, 76.7% attributed the risk of disease at the destination as the main reason for accepting vaccines. A total of 89.3% knew that there was a risk of YF in Brazil. A total of 40% answered correctly only one question about the YF vaccine; 21.6% did not answer any questions correctly. Thus, most of the Brazilian immigrants in this study have high general knowledge about vaccines/vaccination, few have a favorable attitude, and their knowledge about the YF vaccine is scarce. This could limit vaccination adherence when visiting Brazil, making health education actions necessary to increase knowledge and prevent YF risks.

## 1. Introduction

After the advent of sanitation and potable water, vaccination was the most effective phenomenon in preventing infectious diseases, which, for many years, were the main cause of mortality in the world. Advances in the development of vaccines have led to a reduction in the spread of infectious diseases and have created a population that is immune to numerous pathologies preventable by vaccination [1]. The impact of vaccinations on improving the health of populations is undeniable. However, this effectiveness, which is statistically proven over time, does not prevent the existence of groups of people who are still hesitant to receive vaccines and others who support movements against their use [2].

International travelers are potential importers and transmitters of diseases between different regions of the planet. Vaccines are prescribed for travelers based on technical criteria in a pre-travel medical appointment with a doctor or specialist nurse. For some vaccines, more than one dose may be needed to complete the first vaccination, justifying the importance of adequate vaccination in time before travel [3,4,5].

Yellow fever is a hemorrhagic viral infectious disease caused by an arbovirus of the genus *Flavivirus*, whose natural reservoir is non-human primates that inhabit tropical forests. Transmission to non-immunized humans occurs through the bite of infected mosquitoes of the genera *Aedes*, *Haemogogus*, and *Sabethes*, with no direct transmission from person to person. The risk of yellow fever virus infection in unvaccinated travelers is imminent in countries endemic to the disease [6,7]. For a two-week stay in South America, the estimated risk of illness and death due to yellow fever for unimmunized travelers is 5 in 100,000 and 1 in 100,000, respectively [8].

In Brazil, yellow fever outbreaks recently occurred in areas of states previously considered non-endemic, such as São Paulo, Minas Gerais, and Rio de Janeiro. The yellow fever vaccine was not part of the National Immunization Program in non-endemic areas, but it has been recommended since 2017 [9].

Wieten and colleagues demonstrated the lasting effect of protection from the yellow fever vaccine, suggesting that a single dose of the 17D vaccine may be sufficient for long-term protection due to the presence of antibodies with a specific memory for yellow fever up to 18 years later and titters of antibodies at protective levels for the disease for 35 to 40 years after primary vaccination [10].

In Portugal, the international recommendations for vaccination against yellow fever are that all individuals without contraindications to the yellow fever vaccine who are traveling to the endemic area, must have a dose of this vaccine, even if it is not a legal requirement to enter the country [11]. Currently, there are 45 International Vaccination Centers, IVCs (yellow fever vaccination centers), throughout the country, i.e., in mainland Portugal and the autonomous regions of Madeira and the Azores, which are integrated into public primary health care centers, public hospitals, and clinics [11]. The yellow fever vaccine can only be administered at an IVC, and a medical prescription is required. After vaccination, the centers provide a certificate of vaccination. IVCs have a geodemographic area of intervention associated with travelers’ areas of residence. The vaccine is not free and is paid for by the traveler himself. In our country, most travelers do not seek advice before traveling, especially those who go to visit family and friends. Of those who sought advice at a travel clinic, two-fifths came to receive the yellow fever vaccine, which will only be prescribed if they travel to an endemic area; the remaining three-fifths sought advice on vaccination, malaria chemoprophylaxis, and care in the destination country [12].

Studies on knowledge, attitudes, and practices (KAP) are widely used in the health area, contributing to the planning of public health programs. They are commonly used to obtain information about a community that is intended to be studied, measuring the level of knowledge, attitudes, and preventive health practices in the group under study. This methodology is useful for measuring the knowledge acquired after educational actions have been implemented in the community. KAP research is characterized by an easy-to-execute and cost-effective methodology. It also allows easy-to-interpret quantifiable data, in addition to an accurate presentation of results, which could eventually be generalized to a larger population [13,14].

This study aims to characterize the knowledge, attitudes, and aspects of literacy regarding vaccination in general, and particularly vaccination against yellow fever, among Brazilian immigrants in Portugal.

## 2. Material and Methods

A quantitative, observational, descriptive, cross-sectional survey was carried out using a self-completed questionnaire (in person or remotely) to obtain data and assess knowledge and attitudes regarding vaccination among Brazilian immigrants in Portugal. An analytical component was included in the study.

### 2.1. Population and Sample

In Portugal, Brazilian nationality has been the most represented among immigrants, corresponding to around thirty percent of the total number of immigrants in the country (150,864 individuals in 2019; 239,744 individuals in 2022) [15,16].

The inclusion criteria for the study were as follows: being a first-generation migrant with Brazilian nationality, currently residing in Portugal for more than six months, being 18 years old or older, not having cognitive, communication, or mental disorders or other situations that prevent answering the questionnaire, consenting to participate in the study due to availability and willingness, and signing the informed consent form.

The questionnaire was applied in the places most frequented by the Brazilian migrant community in the country: hairdressing salons (male and female), English schools, and evangelical churches in the districts of Évora and Lisbon, and the Consulate General of Brazil in the cities of Lisbon and Porto. In this way, the geographical area of residence of the study participants was made up of migrants living in almost all Portuguese districts.

When calculating the sample size, an estimate of the proportion of 0.5, a desired precision of 0.05, and a confidence level of 95% were considered. Forty percent was added to the value obtained so that the statistical tests maintained applicable conditions if the participants did not answer a question. The intended sample was 539 individuals; the final sample for this study consisted of 542 Brazilian immigrants. Convenience sampling was used.

### 2.2. Data Collection

Participants in the study were recruited from June 2019 to March 2020. A self-completed questionnaire survey was administered in the presence of the researcher or via the Internet. At the Consulate General of Lisbon and Porto, Brazilian migrants were recruited by order of arrival. Recruitment via the Internet was carried out in informal groups of migrants using the WhatsApp messaging application, where the research questionnaire was applied using Google Forms.

### 2.3. Data Collection Instrument

The questions were developed from a literature review of studies on vaccination knowledge and practices. The linguistic and cultural regionalisms of the Brazilian community in Portugal were also considered.

To guarantee the validity of the content of the items that made up the instrument, a consensus methodology (Delphi technique) was carried out through a panel of experts in travel medicine, a method used and accepted to achieve convergence of opinion within a given topic [17]. The initial version of the instrument, developed from a literature review, was reviewed by a panel of thirteen experts, of which nine were Portuguese and four were Brazilian. To be considered experts, professionals should have at least two years of work in travel medicine, in Brazil or Portugal. The experts assessed the relevance of each item that made up the data collection instrument. After the analysis by experts and reaching consensus, the indicated suggestions were inserted, and the questionnaire was redone.

A pre-test was then carried out with a convenience sample of 30 Brazilian immigrants in Portugal to verify the acceptability, understanding, and applicability of the instrument and adjust items and/or response instructions.

The questionnaire contained questions about knowledge and attitudes toward vaccination in general, vaccination for travelers, and vaccination against yellow fever, as well as questions for the socio-demographic characterization of respondents.

### 2.4. Data Processing and Analysis

The data were analyzed with the Statistical Package for the Social Sciences (version 25) for Windows, from IBM SPSS Statistics. A significance level of 5% was adopted for the maximum probability of error in rejecting the null hypothesis when this hypothesis is true. The existence of significant differences was considered whenever *p* < 0.05. The Mann–Whitney test was used as an alternative to the t-Student test for two independent samples when the theoretical assumptions of applicability were not met. The Mann–Whitney test allows comparing medians of variables on at least an ordinal scale. The Kruskal–Wallis test was used as an alternative to the one-way analysis of variance (One-Way ANOVA) when the applicability assumptions were not met. This test is used to check whether *k* independent samples were extracted from populations with the same location (median, for example). The Spearman correlation test was used to test the correlation between pairs of variables where both were ordinal or when one variable was ordinal and the other quantitative.

### 2.5. Ethical Considerations

The protocol was submitted for evaluation and received approval from the Ethics Committee of Instituto de Higiene e Medicina Tropical, Universidade Nova de Lisboa (Institute of Hygiene and Tropical Medicine, NOVA University of Lisbon)—number 02.19.

Participation in the study was voluntary; the information provided by the participants was confidential; and the questionnaires were anonymous. There were no risks of physical or psychological harm when participating in the study (such as embarrassment when answering the questionnaire, discomfort, fear, shame, stress, or tiredness). Participation in the study did not involve any monetary compensation or expense for the respondent.

## 3. Results

### 3.1. Sociodemographic Characterization

The study included 542 first-generation Brazilian immigrants over 18 years old and residing in Portugal for more than six months; 67.9% (368/542) of participants were recruited in person by researchers, and 32.1% (174/542) responded to the same questionnaire online using Google Forms.

Table 1 shows the sociodemographic characteristics of the participants in the study. Participants were aged between 18 and 70 years old, with a mean age of 36.8 years old, a standard deviation of 11.78 years old, and a median of 35 years old. Around a quarter have lived in Portugal for at least ten years, and another quarter for more than a year but less than two. There was a predominance of people who lived in the southeast region of Brazil, mainly in the States of Minas Gerais (19.8%), São Paulo (17.0%), and Rio de Janeiro (10.4%), for a total of 47.2% (100/212). 17.5% (37/212) of respondents lived in Goiás, a state in the center-west region of Brazil, the second most represented state.

### 3.2. General Knowledge about Vaccines

**Figure 1:**
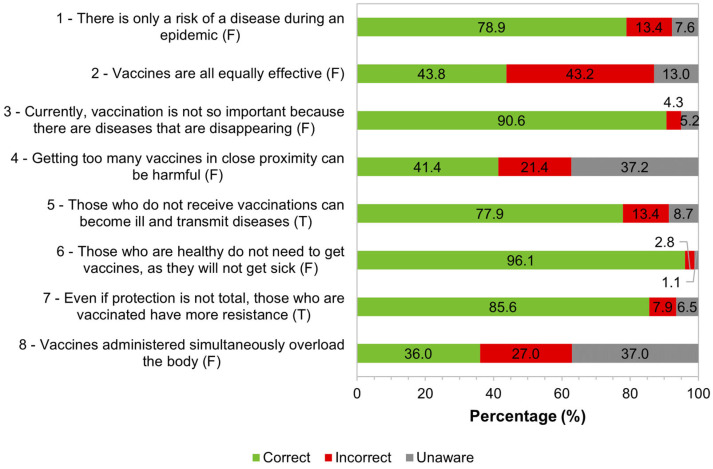
Distribution of responses regarding general knowledge about vaccines/vaccination. Correct answer: False (F); True (T). 1: *n* = 536; 2: *n* = 537; 3: *n* = 541; 4: *n* = 541; 5: *n* = 539; 6: *n* = 540; 7: *n* = 541; 8: *n* = 541.

To characterize general knowledge about vaccines/vaccination, several statements were presented with the answer options “True”, “False”, and “Unaware”. For each sentence, a correct answer was considered when the study participants answered “False” in false statements or when they answered “True” in true statements. The distribution of answers can be seen in Figure 1.

The majority of participants demonstrated that they knew that there is a risk of contagion of a disease regardless of the occurrence of an epidemic and recognized that vaccination is important to prevent diseases. Most participants also considered that unvaccinated individuals could become ill and transmit infectious diseases to other people; however, 13.4% (72/539) did not believe in this possibility. Regarding the need for vaccination while being healthy, the overwhelming majority considered it necessary for all individuals, healthy or not, believing there was a risk of any non-immunized individual becoming ill. Regarding the simultaneous administration of multiple vaccines, there was a balance between the percentage of participants who did not know and those who responded that it was false that the administration of multiple vaccines overloads the body. It should be noted that 27% (146/541) considered that the simultaneous administration of multiple vaccines overloads the body and that 21.4% (116/541) considered that having several vaccines on close dates could be harmful (Figure 1).

**Figure 2:**
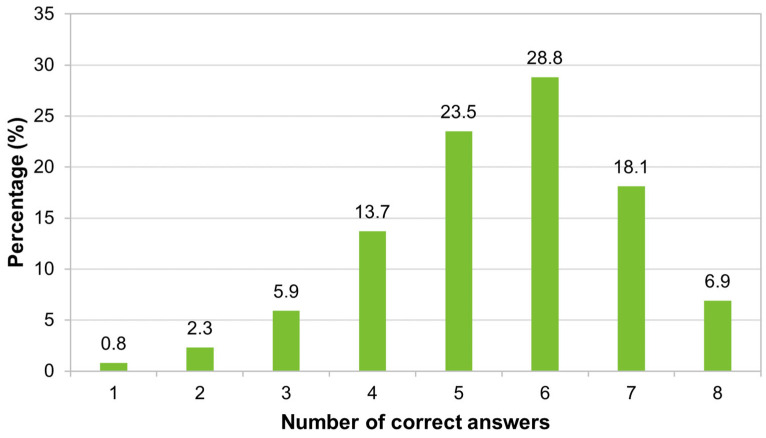
Distribution of respondents according to the number of correct answers about vaccines/vaccination (*n* = 524).

Regarding the number of correct answers about vaccines/vaccination, more than half of the participants (53.8%, 282/524) answered at least six questions correctly, but only 6.9% (36/524) correctly answered all the presented questions (Figure 2).

There was a significant difference between genders in the number of correct answers to general questions about vaccination, with a larger number of correct answers among female participants (mean rank 273.99) than among male participants (mean rank 244.69) (Mann–Whitney test, U = 293558.500, *p* = 0.026). There was a positive correlation between the level of education and the number of correct answers (Spearman test, r_s_ = 0.305, *p* < 0.001) and a negative correlation between the number of years in Portugal and the number of correct answers (Spearman test, r_s_ = −0.141, *p* = 0.001). No significant correlation was found between the respondents’ age and the number of correct answers (Spearman test, *p* = 0.329).

### 3.3. Attitudes toward Vaccination

**Figure 3:**
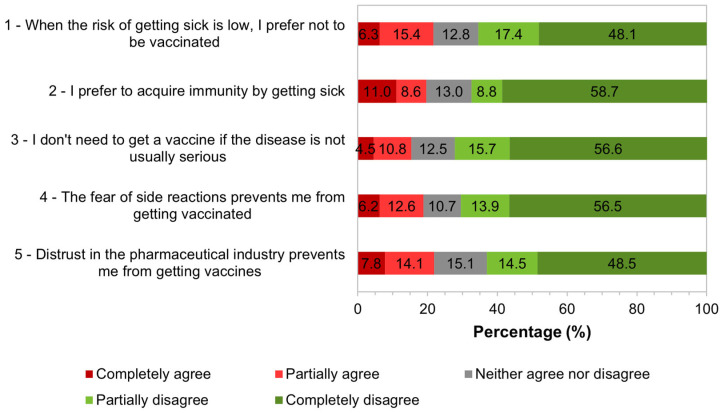
Distribution of respondents according to the degree of agreement or disagreement with the statements. 1: *n* = 539; 2: *n* = 537; 3: *n* = 530; 4: *n* = 531; 5: *n* = 524.

Attitude is a “latent variable”, i.e., a variable that cannot be observed or measured directly but that can be defined based on other variables that can be observed/measured. Therefore, participants were asked to indicate their degree of agreement or disagreement with the statements proposed in the questionnaire (Figure 3). It was considered that respondents who disagreed with a statement had an attitude in favor of vaccination.

The responses of each immigrant to all statements presented in Figure 3 were simultaneously considered. It was considered that anyone who responded that they completely disagreed with all statements had a “Totally favorable attitude” toward vaccination; those who completely agreed had a “Totally unfavorable attitude” toward vaccination; those who responded “Neither agree nor disagree” had a “Totally indifferent attitude”; those who responded that they completely agreed or partially agreed tended to have an “Unfavorable attitude”; and those who responded that they partially disagreed or completely disagreed tended to have a “Favorable attitude”. Those who did not follow these response patterns were classified as having an “Undefined attitude”.

**Figure 4:**
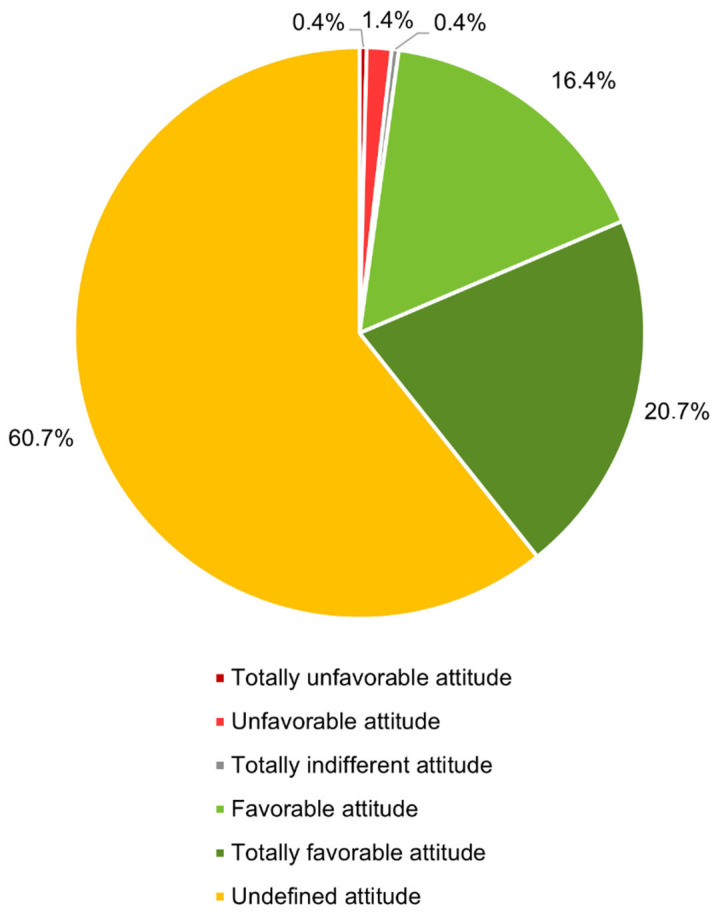
Distribution of respondents according to their attitude toward vaccination (*n* = 517).

In this way, we were able to determine that the majority of participants in the study (60.7%, 314/517) did not have a defined attitude toward vaccination, while 20.7% (107/517) showed a completely favorable attitude toward vaccination (Figure 4). Overall, 37.1% (192/517) tended to be favorable to vaccination, and only 1.8% (9/517) tended to be unfavorable to it.

### 3.4. Knowledge about Vaccination for Travelers

**Figure 5:**
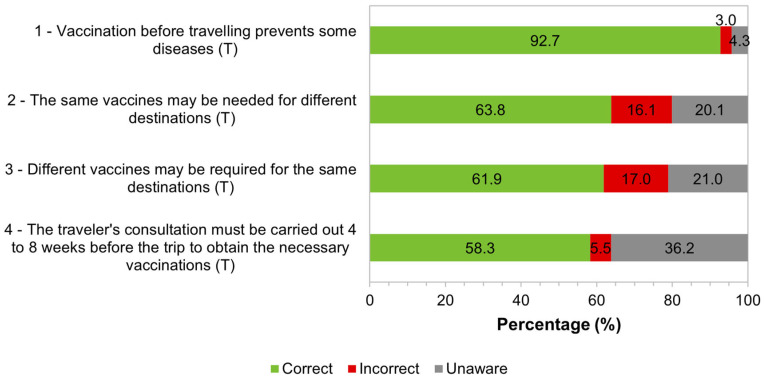
Distribution of responses regarding knowledge about travelers’ vaccination. Correct answer: True (T). 1: *n* = 532; 2: *n* = 528; 3: *n* = 528; 4: *n* = 530.

Participants demonstrated their knowledge by answering “True”, “False”, or “Unaware” to the sentences presented. For each sentence, a correct answer was considered when the participants in the study answered “True” to the statements in the questionnaire. The distribution of answers can be seen in Figure 5. The majority demonstrated that they were aware of the importance of vaccination in preventing diseases in international travelers (92.7%, 493/532); they were also aware of the fact that the same vaccines may be recommended for different destinations (63.8%, 337/528); they considered that different vaccines may be necessary for travelers traveling to the same destination (61.9%, 327/528); and that the traveler’s consultation should be carried out four to eight weeks before the trip (58.3%, 309/530).

**Figure 6:**
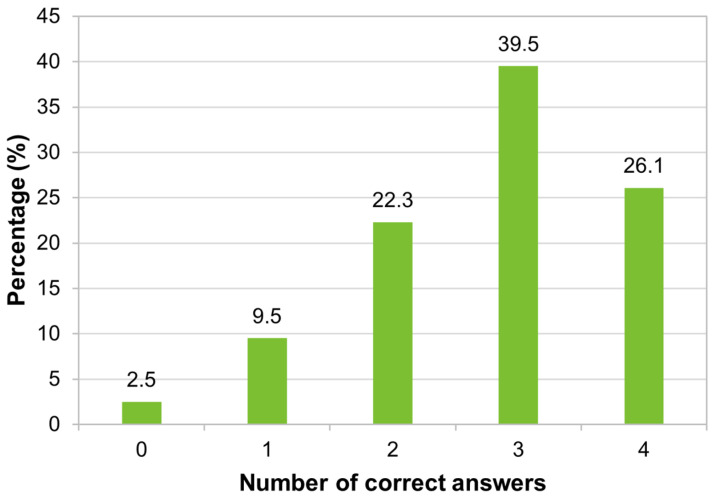
Distribution of respondents according to the number of correct answers about vaccines for travelers (*n* = 524).

It was found that the largest percentage of participants in the study (39.5%, 207/524) correctly answered three-quarters of the asked questions, but 2.5% (13/524) of respondents did not correctly answer any question on the topic (Figure 6). No difference was found between genders (Mann–Whitney test, *p* = 0.907), nor was there a correlation between age and the number of correct answers about vaccines for travelers (Spearman test, *p* = 0.244). There was a negative correlation between the number of correct answers and the number of years of residence in Portugal (Spearman test; r_s_ = −0.108, *p* = 0.015) and a positive correlation with the level of education (Spearman test; r_s_ = 0.092, *p* = 0.036).

The Brazilian immigrants who participated in the study indicated that it was very important for greater acceptance of vaccination: trust in the doctor’s recommendations (75.1%, 396/527), the excellent protective effect of the vaccine (64.8%, 339/523), and the risk to which they are exposed at the travel destination (76.7%, 402/524).

### 3.5. Knowledge about Yellow Fever and the Yellow Fever Vaccine

**Figure 7:**
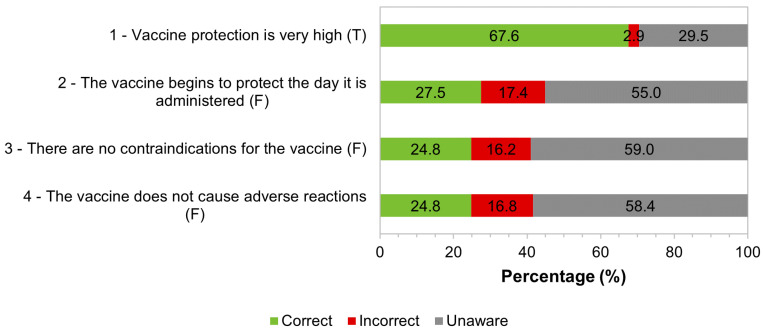
Distribution of respondents regarding knowledge about the yellow fever vaccine. Correct answer: False (F), True (T); 1: *n* = 516; 2: *n* = 516; 3: *n* = 517; 4: *n* = 517.

In order to assess knowledge about yellow fever and the yellow fever vaccine, immigrants answered “True”, “False”, or “Unaware” to the statements. For each sentence, a correct answer was considered when the participants in the study answered “False” in false statements, or when they answered “True” in true statements. The distribution of answers can be seen in Figure 7.

The majority of the participants in the study (89.3%, 478/535) answered correctly that there is a risk of the disease in Brazil, but 8.2% (14/535) were unaware of this risk. The majority (67.6%, 349/516) demonstrated that they knew that the degree of protection of the vaccine against yellow fever is very high; 55% (284/516) were unaware of the period necessary to develop protection after vaccination; 59% (305/517) were unaware of whether there were contraindications to administering the vaccine; and 58.4% (302/517) were unaware of whether there could be adverse reactions (Figure 7).

**Figure 8:**
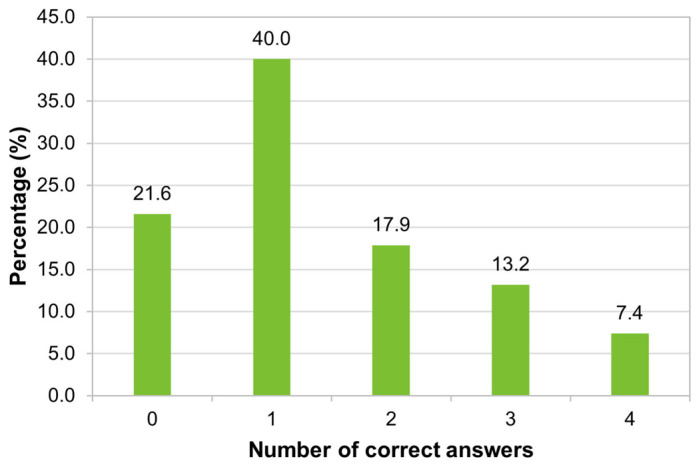
Distribution of respondents according to the number of correct answers about the yellow fever vaccine (*n* = 515).

The largest percentage of participants in the study (40%, 206/515) answered only one of the four questions correctly, and 21.6% (111/515) did not answer any of the questions correctly. Only a small percentage (7.4%, 38/515) answered all of the questions correctly (Figure 8).

There was no significant difference between genders regarding the number of correct answers about the yellow fever vaccine (Mann–Whitney test, *p* = 0.725), correlation with age (Spearman test, *p* = 0.147), or relationship with having lived or not in a yellow fever endemic area (Kruskal–Wallis test, *p* = 0.915). There was a positive correlation between the level of education and the number of correct answers to questions about the yellow fever vaccine (Spearman test; r_s_ = 0.227, *p* < 0.001) and a negative correlation with the length of residence in Portugal (Spearman test; r_s_ = −0.176, *p* < 0.001).

## 4. Discussion

This research made it possible to get closer to the real knowledge and attitudes regarding vaccination among Brazilian immigrants in Portugal.

Regarding sociodemographic characteristics, study participants were relatively young, with more than half (51.7%) aged between 18 and 35 years. Most of them were educated individuals, with a high percentage (44.7%) of people with a Bachelor’s, Master’s, or Doctorate degree. Forty-five percent of respondents have lived in Portugal for more than three years, having immigrated before 2017, the year in which vaccination against yellow fever began to be recommended in new regions of several states in Brazil (Bahia, Espírito Santo, Rio de Janeiro, São Paulo, Paraná, Santa Catarina, and Rio Grande do Sul) because of a large yellow fever outbreak in those states. Therefore, it is likely that a large number of immigrants who lived in these states (45.3%, 96/212) have never been vaccinated against yellow fever.

The majority of participants (77.3%) had a high level of knowledge about vaccination in general, answering correctly more than three-quarters of the eight questions proposed. Only a minority (9%) had low knowledge of this topic. Regarding the simultaneous administration of multiple vaccines, only a little more than a third of respondents knew that this practice does not cause harm to the body, and around two-thirds were unaware or answered incorrectly. Almost all questions (Figure 1) presented a higher percentage of answers in the “Correct” category than in “Incorrect” or “Unaware”. The highest percentages of “Unaware” responses were found in questions four and eight, while the highest percentages of answers in the “Incorrect” category were in questions two and eight. These responses may be related to a lack of technical knowledge, and, despite the high level of education of the participants in the study, their training may not be related to the health area.

In a similar study carried out to assess knowledge and attitudes about vaccination [18], it was shown that the majority of participants considered vaccines efficient and safe to prevent diseases, 93% and 84%, respectively. In another study on knowledge about vaccines [19], the majority of participants believed that vaccines were effective, but 15.3% considered them to be unsafe. Other authors showed that vaccines were considered unnecessary and unsafe by a growing number of people who had an anti-vaccination attitude and refused to be vaccinated [20]. It was also found that, although the majority of participants considered vaccination to be effective, almost 10% did not consider it important for disease prevention [21]. In a study that measured the knowledge and attitudes of travelers regarding vaccination [22], a similar result was found concerning the need to administer vaccines to all people, with it being observed that 67% were aware of the need to administer vaccines to all travelers. However, knowledge of vaccine-preventable diseases was scarce. Parents’ concerns about their children’s vaccinations were noted [23]: 34.2% reported fear of administering too many vaccines during a single appointment, in addition to other concerns such as pain and the emergence of other diseases. However, in the indicated study, around 80% believed that vaccines were important and were confident in using them in their children.

In our study, perhaps the larger number of correct answers among female individuals may be related to the fact that women take their children to the doctor more frequently. The greater number of correct answers among migrants with a higher level of education may be related to greater access to information about health and vaccination.

It was observed that a high percentage of the studied sample, around 60%, had an undefined attitude toward vaccination. This percentage was higher than expected since each statement corresponded to a “belief”. It would be expected that anyone who was in favor of vaccination would disagree with all the statements, and vice versa. Among the participants who showed a definite attitude, the majority had a favorable attitude toward vaccination. This represents important data since the way of thinking plays an important role in deciding whether or not to accept vaccines.

Several studies [18,19] have shown that the fear of adverse side reactions is among the main causes cited for vaccine hesitancy, in addition to the fear of pain and/or needles and the belief that vaccines are unnecessary. The main reasons for fear of vaccination were concerns about the safety of vaccines, the unreliability of the way they are manufactured, and the fear of side effects [19].

Our study revealed a high level of knowledge regarding vaccination for travelers: around two-thirds of the studied migrants answered correctly more than 75% of the four questions proposed about vaccination for travelers. Most were aware that vaccination before travel can reduce the risk of illness and that vaccines may be necessary according to the specific characteristics of each traveler-travel type pair and not just regarding the destination location. However, many were unaware that the traveler’s consultation must be carried out 4 to 8 weeks before the trip to receive the necessary vaccination. The results indicate that greater awareness is needed about the importance of travel advice.

Some studies revealed that non-immune international travelers are, in general, responsible for the importation of vaccine-preventable diseases, from endemic, emerging, or re-emerging diseases existing in limited areas to universal pathologies [24]. Other studies, which assessed knowledge, attitudes, and health practices while traveling, demonstrated the deficiency in knowledge and preventive practices of travelers about travel-associated infectious diseases, in addition to the need to increase traveler awareness [25]. It was also found that among travelers/migrants visiting friends and relatives, only 13% were aware of the need for vaccination [25]. A study at Spanish airports found that the majority of travelers (73%) sought medical advice an average of 19 days before traveling and that more than half of travelers heading to areas at risk of infectious diseases did not receive vaccines [21]. In Lisbon, Portugal, it was shown that, among travelers who sought consultations before traveling, only around half used a specialized service in travel medicine [26]. A study at Australasian airports (Singapore, Kuala Lumpur, Taipei, Melbourne, and Seoul) whose travel destinations were Asia, Africa, or South America, highlighted the educational need to encourage greater acceptance of health advice before traveling [27]. Confidence in the doctor’s recommendation, the severity of the disease, and personal and family protection were the most important reasons for vaccination [18]. Pre-travel counseling carried out by doctors trained and experienced in travel medicine contributed to greater acceptance of vaccines recommended for international travelers [25], and being in favor of vaccination is related to the excellent protective effect that the vaccine can provide against the disease [28]. Acceptance of vaccines by travelers is associated with high endemicity and the risk of vaccine-preventable disease [25,29]. These last studies were in agreement with ours since we also observed that trust in the doctor’s recommendations, the excellent protective effect of the vaccine, and the risk of disease at the travel destination play an important role in the acceptance of vaccination.

In our study, a high percentage (89.3%) of migrants knew that there is a risk of acquiring yellow fever in Brazil, but for 8.2%, yellow fever does not represent a risk of infection in the country. The Brazilians surveyed had low knowledge regarding issues related to the yellow fever vaccine, as two-fifths answered correctly only one of the four questions asked on the topic, and around one-fifth got all the questions wrong. The low knowledge observed is justified due to the need for technical knowledge in health for a better understanding of these characteristics of the vaccine. Despite the majority of respondents having a Bachelor’s degree, a Master’s degree, or a Doctorate, their training may not be related to specific knowledge in the health area. The larger number of correct answers from immigrants surveyed with shorter residence times in Portugal may be related to the fact that they remember that the yellow fever vaccine is recommended for endemic areas of Brazil or because they were vaccinated in the region where they lived. In Brazil, vaccines against measles, yellow fever, and influenza were the best known, followed by vaccines against hepatitis B, tetanus, rubella, and mumps [19].

Although Brazil is a country with a risk of yellow fever transmission, it does not require this vaccine for travelers arriving in the territory. The legal requirements for entering a country are defined by the competent authorities of the country itself. Competent entities in their countries will be able to develop strategies so that travelers/migrants from their territory adopt preventive behaviors in the countries to which they are going to travel.

To increase the level of knowledge on the topics studied in our research, as well as the percentage of Brazilian immigrants with a favorable attitude toward vaccination, some appropriate information techniques could be used in the places most frequented by Brazilian immigrants: distribution of leaflets, lectures, or videos in immigrant associations; distribution of leaflets in places most frequented by this community; messages transmitted through social networks. The sources/senders of these messages should be individuals/institutions with prestige/credibility among these immigrants. The role of peers in disseminating vaccination education messages is also very important.

Although all the proposed objectives were met, some aspects limited the completion of this work. It is worth highlighting: the use of a convenience, non-random sample does not allow extrapolation of the results to the entire Brazilian community in Portugal; the difficulty in recruiting Brazilian immigrants, due to the lack of time to answer the questionnaire or the fear of being exposed in the case of immigrants with an irregular situation in Portugal (even guaranteeing anonymity and confidentiality; the lack of economic resources necessary to travel to several cities with a larger population of Brazilians to better recruit participants; the start of the COVID-19 pandemic prevented greater contact with individuals; and the non-response (missing) to some questions limited the data analysis/conclusions on some topics. It has been suggested that the COVID-19 pandemic highlighted the relevance of vaccines to many people who trust vaccination but may have negatively affected those who were hesitant or misinformed. Thus, if the study had been carried out after the pandemic, even if the same people had been recruited, the results on general knowledge and attitudes toward vaccination could have been different due to the broad debate on vaccines that took place at that time. However, it is unlikely that knowledge about vaccines for travelers and knowledge about the yellow fever vaccine would have been different because of the specificity of the questions asked.

## 5. Conclusions

The highest number of correct answers about vaccination was found in Brazilian immigrants with a higher level of education and in those who have resided in Portugal for a shorter time. In the case of general knowledge about vaccines, women showed more knowledge than men. In this study, more than half of the participants did not have a defined attitude toward vaccination, a third had a generally favorable attitude, and a minority had an unfavorable attitude toward vaccination, which could limit adherence to the necessary vaccination when traveling internationally. Brazilian immigrants in Portugal who immigrated more than 10 years ago or came from states where there was no risk of yellow fever may never have had this vaccine. It is necessary to reinforce educational interventions in the Brazilian migrant community in Portugal in order to increase knowledge about the characteristics of the vaccine/vaccination against yellow fever.

## Figures and Tables

**Table 1:** Sociodemographic characteristics of study participants.

Attribute		*n*	%
Age (years) *n* = 535	18–25	94	17.6
	26–35	183	34.1
	36–45	137	25.7
	46–55	71	13.2
	56–65	44	8.2
	66–75	6	1.2
Gender *n* = 541	Male	217	40.1
	Female	324	59.9
Education/years of schooling *n* = 535	Did not study	2	0.4
	≤4 years	17	3.2
	(4–9) years	35	6.5
	(9–12) years	240	44.8
	Bachelor’s degree	171	32.0
	Master’s/Doctorate degree	68	12.7
	Other	2	0.4
Residence in Portugal (years) *n* = 519	>6 months and <1 year	89	17.1
	(1–2) years	135	26.0
	(2–3) years	62	11.9
	(3–4) years	34	6.6
	(4–5) years	19	3.7
	(5–10) years	40	7.7
	≥10 years	140	27.0
Residence in Brazil *n* = 212	Yellow fever endemic zone	206	97.3
	Non-endemic zone	6	2.7

## Data Availability

The data presented in this study are available on request from the corresponding author due to privacy reasons.
